# Animal evolution and atmospheric pO_2_: is there a link between gradual animal adaptation to terrain elevation due to Ural orogeny and survival of subsequent hypoxic periods?

**DOI:** 10.1186/1742-4682-11-47

**Published:** 2014-10-22

**Authors:** Sven Kurbel

**Affiliations:** Osijek Medical Faculty, Department of Physiology, J Huttlera 4, 31000 Osijek, Croatia

**Keywords:** Isolated habitats, P-Tr extinction, K-Pg extinction, Devonian extinction, Amphibian evolution, Insect evolution, Archosaur evolution, Mammalian sex chromosomes

## Abstract

Considering evolution of terrestrial animals as something happening only on flat continental plains seems wrong. Many mountains have arisen and disappeared over the geologic time scale, so in all periods some areas of high altitude existed, with reduced oxygen pressure (pO_2_) and increased aridity. During orogeny, animal species of the raising terrain can slowly adapt to reduced oxygen levels.

This review proposes that animal evolution was often driven by atmospheric oxygen availability. Transitions of insect ancestors and amphibians out of water are here interpreted as events forced by the lack of oxygen in shallow and warm water during Devonian. Hyperoxia during early Carboniferous allowed giant insects to be predators of lowlands, forcing small amphibians to move to higher terrains, unsuitable to large insects due to reduced pO_2_. In arid mountainous habitats, ascended animals evolved in early reptiles with more efficient lungs and improved circulation. Animals with alveolar lungs became the mammalian ancestors, while those with respiratory duct lungs developed in archosaurs. In this interpretation, limb precursors of wings and pneumatised bones might have been adaptations for moving on steep slopes.

Ural mountains have risen to an estimated height of 3000 m between 318 and 251 Mya. The earliest archosaurs have been found on the European Ural side, estimated 275 Myr old. It is proposed that Ural orogeny slowly elevated several highland habitats within the modern Ural region to heights above 2500 m. Since this process took near 60 Myr, animals in these habitats fully to adapted to hypoxia.

The protracted P-Tr hypoxic extinction event killed many aquatic and terrestrial animals. Devastated lowland areas were repopulated by mammaliaformes that came down from mountainous areas. Archosaurs were better adapted to very low pO_2_, so they were forced to descend to the sea level later when the lack of oxygen became severe. During the Triassic period, when the relative content of O_2_ reduced to near 12%, archosaurs prevailed as only animals that could cope with profound hypoxia at the sea level. Their diverse descendants has become dominant terrestrial animals, until the K-Pg extinction due to meteor impact.

**Electronic supplementary material:**

The online version of this article (doi:10.1186/1742-4682-11-47) contains supplementary material, which is available to authorized users.

## Introduction

General process of animal life adaptations to an ever-changing planet cannot be understood without a complex interplay of animal physiology and prevailing climate features. Extremely slow climate changes, such as orogeny due to plate tectonics also need to be incorporated. This combined approach is analogous to the “escalation hypothesis” [1], based on the idea that successful species with traits that keep them numerous and viable under static conditions, can become more endangered during the dynamics of an extinction event then some marginal and less adapted animals. A consequence is that ecological opportunity probably played a role at certain points along the lineage of mammals [2].

In a recent theoretic paper [3], a hypothesis is put forward that the occurrence of homeothermy can be understood only consequently of a long evolution process that lasted more than 50 Myr. Here presented interpretation goes further in that direction, with the idea that during several periods of animal evolution altered by hypoxic events, animals adapted to high altitude habitats could survive through migration to areas closer to the sea level. Similar to the recently published hypothesis considering the K-Pg extinction [3], here presented interpretation is based on the assumption that all physiological traits need long time to evolve and nothing happens in few generations. If no animal of a certain species has the required surviving feature, there will probably be no survivors.

## Basic assumptions behind the proposed interpretation

Considering evolution of terrestrial animals as something happening only on flat continental plains seems wrong. Due to continuous orogeny, many mountains have arisen and disappeared over the geologic time scale, so in all geologic periods some areas are near the sea-level, while other areas are much higher. Although the O_2_ volume percent remains the same, areas of higher altitudes are under lower oxygen pressure (pO_2_) and usually less humid. This means that during any significant orogeny, animal species of the raising terrain can slowly adapt to reduced oxygen levels. This setting allows parallel evolutions of similar animals in habitats separated by altitude, leading to a stratified biosphere with different animal worlds at the sea level, in low or in high mountains.

Beside changes in CO_2_ levels, two great extinctions seem directly related to the altered oxygen availability. The older is the late Devonian extinction period ending with a hypoxic Hangenberg event (est. 358.9 ± 0.4 Mya) that has badly affected marine and terrestrial habitats and left an anoxic black shale layer with overlying sandstone deposits [4]. The more recent is the large Permian–Triassic extinction event (some 251 Mya), so severe that the animal world required millions of years to recover.

Beside these abrupt hypoxic extinctions, animal evolution was also affected by slow accumulation of atmospheric oxygen, particularly during Carboniferous period when combination of high O_2_ and low CO_2_ resulted in cooler and drier climates.

## The atmospheric O2 pressure driven animal evolution

### Insects as first terrestrial animals

Terrestrial life started with first plants near the coastal line. At that period, most of the oxygen was produced by the plant life in ocean were all animals lived. The atmospheric O_2_ level was low and oxygen availability depended almost entirely on diffusion from the ocean.

Insects, as the first terrestrial animals are assumed to have developed from early Crustaceans during early to middle Devonian, probably more than 400 Mya [5]. It can be only speculated what made insects the first terrestrial animals. The first step might have happened when some Crustacean survived in an isolated lake of increased acidity by using pure chitin in building exoskeletons. These early ancestors of insects were forced to remain in water up to the moment when early terrestrial plants have become so abundant to act as a new important source of oxygen. Then the atmospheric O_2_ started to diffuse into the superficial water layer, allowing ancestors of insects to dwell near the water surface. Oxygen abundance and lack of air breathing predators made these small and lightweight creatures terrestrial animals. This process must have taken many generations until some of them became able to move on the water surface, or to breathe air through their thin exoskeletons. Their descendants have developed small holes in the chitinous exoskeleton, so the formation of tracheas was probably the next step toward larger and more capable insects.

### The late Devonian phase: hypoxia and emergence of teraphods

The late Devonian extinction event is possibly related to the development of teraphods. Before the extinction, the land near the coast line and along rivers had been already colonized by plants and insects. When insects invaded land in Devonian, the average O_2_ air content was near 16%. Since only small insects survived the Triassic nadir with protracted periods of only 12% of oxygen [6, 7], it can be assumed that the share of atmospheric oxygen during the Hangenberg event was probably also somewhere between 16% and 12%.

It is generally assumed that the amphibians developed in the Devonian period, around 370 million years ago. They came from earlier lobe-finned fish similar to the modern coelacanth and lungfish, which had evolved multi-jointed leg-like fins with digits that enabled them to crawl along the shallow bottom [8–10].

The important examples of the early amphibian evolution include:

- ! Canada
  " *Eusthenopteron foordi* lived during the Late Devonian period, about 385 million years ago. The fossils have been found in Miguasha, Quebec [11].
  " *Tiktaalik roseae* is an extinct lobe-finned fish from the late Devonian period, 383-million-year-old, found on Ellesmere Island in Nunavut, Canada [9]
  " *Elpistostege watsoni* is an extinct tetrapod-like fish, found at Escuminac Formation in Quebec, Canada [12].
- ! East Greenland
  " *Ichthyostega stensioei* lived at the end of the Upper Devonian period (374 – 359 million years ago), with developed lungs and limbs [13].
- ! Europe
  " Fossils of *Panderichthys rhombolepis*, an extinct fish with differentiated distal radial bones, were found in Baltic sediments of Latvia and Poland [14], estimated as 397 million years old.
- ! Western Australia
  " Fossils of *Gogonasus andrewsae*, an extinct 380 million-year-old lobe-finned fish with large *spiracular* openings, found in Western Australia [15].

Noting that all these fossils from nowadays geographically remote locations had similar climates in the late Devonian is important. Due to plate tectonics, some 380 Mya, Ellesmere Island, Quebec and Greenland were all parts of the continent Laurentia. Sometime between 416 and 359.2 Mya, Laurentia collided with Baltica, forming a minor supercontinent Euramerica [16]. So during the critical phase of tetrapod evolution, the eastern Canada, Greenland and Baltica were all near the equator and had a warm and humid climate with abundant bodies of shallow water. Western Australia was then within the Gondwana continent, in the south temperate latitudes, also with a warm and humid climate.

During the introduction of amphibians in the late Devonian [17], the atmospheric O_2_ levels were only 15% [6, 7]. Since O_2_ solubility is reduced in warm water (Additional file 1: Table S1 based on data from: http://www.engineeringtoolbox.com/oxygen-solubility-water-d_841.html), shallow or stationary aquatic habitats can easily become oxygen depleted on a hot day, despite the oxygen abundance in the surrounding air:

! Within this frame of survival pressures, gradual emergence of late Devonian lobe-finned fishes seems inevitable. To get some oxygen from air by using their vascularized gas bladders, their ancestors could not afford much energy expenditure to remain just beneath the water surface. Animals with strong fins could support them near the water surface without muscle activity.

" Breathing air through large *spiracles* while supported by strong fins was advantageous in oxygen depleted waters due to high daily temperatures.

" The next big step was development of primitive lungs, as found in the extant lung fish [18]. Primitive lungs allowed prolonged survival during drought.

Despite primitive lungs and strong, maneuverable fins, no real pressure for fish to leave water and move on land existed before the hypoxic Hangenberg event (some 358.9 ± 0.4 Mya) (Additional files 1 and 2: Tables S1 and S2).

All aquatic animals faced danger from hypoxia and many species vanished. However, the lobe-finned fish had no alterative, but to breathe air and use limbs to move over the land and find better habitats.

A related scenario possibly happened in the sea. Although for more than 100 Myr the global ocean remained warm, it contained sufficient oxygen to support diverse animal life only in the superficial layer [19]. Overall hypoxia has probably forced animals from deeper layers to ascend closer to the water surface.

The proposed interpretation is that terrestrial migration of early amphibians should be considered as an inevitable event caused by the hypoxic Hangenberg event.

### The Carboniferous phase of animal evolution under oxygen abundance

Since the first terrestrial plants were quickly followed by early insects, it can be assumed that early insects ate either plants, or other insects. Indirect evidences that the Carboniferous world was terrorized by early insects can be found in thick bark, small leaves and high lignin content of these early plants. These features prevailed until the P-Tr extinction event that also marks the disappearance of giant insects. Soon after the P-Tr event, the low lignin conifer trees started to spread [20], suggesting that lignin armor was no more necessary to deal with the surviving small Triassic insects.

During the Carboniferous period (358.9–298.9 Mya), the average O_2_ atmospheric content was 32%, while CO_2_ was 800 ppm. It is assumed that the average temperature was similar to present values. During the following Permian period (298.9–252.2 Mya), the average O_2_ content has dropped to 23%, CO_2_ raised to 900 ppm and temperature was 2°C above the present level [6, 7, 21–23].

Amphibians that started to roam the coasts of early Carboniferous rain forests were soon faced with a growing threat from many insects that gradually became giant, due to increasing atmospheric O_2_ content [23] (Additional file 3: Table S3, calculated by the pO_2_ calculator available at: http://www.altitude.org/air_pressure.php).

This danger left the amphibians few choices: some amphibians have also become giant and thus more able to defend from dangerous insects, the other amphibians were forced to migrate along rivers to higher terrains with fewer giant insects. The proposed slow ascend of small amphibians along rivers probably took millions of years, until they have reached the highest mountain slopes with enough water for their survival. The altitude was probably between 1000 and 2000 m above the sea level (Additional files 2 and 3: Tables S2 and S3).

Even during the hyperoxic periods, insects not much larger than the modern insects can live in high mountains [24], due both to low pO_2_ (Additional file 3: Table S3) and reduced humidity [25]. Small amphibians that migrated to high mountains became insectivores of these much smaller insects, instead of being the prey of giant insects at the sea level.

### Diversification of amphibians in high altitude habitats

The amphibians wet skin used for respiration soon became an obstacle for living in more arid high altitude habitats. This was a strong survival pressure for developing impermeable skin and improved lungs leading to early reptiles, able to roam dry mountains and lay amniotic eggs with hard shells. These animals required strong muscles with high perfusion rates to move up the steep mountain slopes. To avoid pulmonary edema due to increased pressures in pulmonary vessels, they must have evolved some type of efficient separation of low pressure pulmonary and high pressure systemic circulation [25, 26]. This development will finally lead to 4-chambered found in avian and in mammalian hearts.

Early reptiles developed more efficient lungs probably of two separate types:

! alveolar lungs developed in reptiles that will become the mammalian ancestors

! lungs with respiratory ducts [27] developed in reptiles that will become archosaurs. Some of these animals remained near the high mountain waters, developing in crocodile ancestors [28].

Overpopulation of mountain water-reach oases made some animals to go deeper into the mountain, looking for food. Most of the archosaurs were heavy built terrestrial animals, thus destined to become dinosaurs in the Jurassic.

The rest of smaller species survived through fast climbing of steep and arid high mountain slopes. They developed light pneumatised bones and, with highly efficient cardiorespiratory system, became the ancestors of pterosaurs and birds. In this interpretation, limb precursors of wings might have been an important adaptation for balancing while moving on steep slopes, particularly during descending.

### Stratification of the biosphere by altitude

It is here proposed that the Carboniferous terrestrial life was stratified in TWO ISOLATED WORLDS:

! at the sea-level, giant insects and amphibians fought in Carboniferous rainforests, while the atmospheric O_2_ content was near 35% [6, 7].

! highland areas formed an archipelago of mountainous zones free of giant animals. These areas were isolated by the low lands rainforests:

" Mountain oases with smaller plants, less water and no threat of giant insects made them probable cradles of animal and plant evolution. This has led to the emergence of rapid growing, thin bark conifers, like the earliest conifers of North America found in the upland [20].

Carboniferous combination of a rise in O_2_ level and drop in CO_2_, has probably resulted from large carbon sequestration when terrestrial plants were buried in swamps and other soft terrains. This lack of plant decomposition resulted in abundant coal deposits. This carbon sequestration is possibly responsible for the gradual global cooling and collapse of Carboniferous rain forests. In the late Carboniferous, climate has become colder, more arid and the relative oxygen content started to decline from the peak value [6, 7]. All these changes made animal life more difficult, particularly for the large insects and amphibians and for animals that lived in mountains.

### Ural orogeny and Permian phase of animal evolution

Noting that during Permian and Triassic, the two important mountain ridges have slowly risen is important. The Australian Hunter-Bowen orogeny started in Permian was in that period far south from the equator. Ural mountains have risen to an estimated height of 3000 m [29] in the northern zone. This orogeny started from 318 Mya, in the late Carboniferous, and lasted to 251 Mya at the P-Tr event. Even today, this old mountain range peaks at 1895 m above the sea level. The earliest known fossils of Archosaurs have been found on the European Ural side, estimated 275 Myr old [30].

It is here presumed that the Ural orogeny slowly elevated several highland habitats within the modern Ural region to heights above 2500 m. Since this process took near 60 Myr, animals in these habitats had enough time fully to adapt to hypoxia (at 2500 m, the pO_2_ is below 80% of the sea level value, Additional file 3: Table S3).

During Ural orogeny, animals could descend to the less elevated mountain slopes that remained above the dangerous lowland habitat. Mammalian ancestors probably remained in many areas bellow 1500 m, with sufficient water and pO_2_ to support limited capacity of their lungs and kidneys, while the early archosaurs with respiratory ducts in their lungs could easily adapt to hypoxia in higher mountain areas (more than 2000 m above the sea level).

### Lignin decomposition as a new biological corrective of climate changes

Perhaps the most striking new peace of evidence, potentially related to these changes in O_2_ and CO_2_ air levels, came from new insights in the lignin decomposition. Floudas et al. [31] analyzed 31 fungal genomes to find out when lignin decomposition had arisen. The results suggest that rot fungi evolved almost simultaneously with a sharp decrease in the rate of organic carbon burial at the end of the Carboniferous period, at around 290 Mya. The decomposition of accumulated and still air exposed lignin probably continued the whole late Permian, since the real “coal gap” happened during the Early Triassic [32] with almost no coal deposits.

Since all rotting uses oxygen and produces CO_2_, it seems plausible that fungal rotting has decomposed accumulated plant material that has not been deeply buried and this carbon was slowly released back into the air as CO_2_. The expected consequence is that lignin rotting has reduced the Permian O_2_ levels and increased the CO_2_ level, leading to the warmer Permian climate.

### Animal migration to lower habitats during the hypoxic P-Tr extinction

The protracted P-Tr hypoxic extinction event with combination of increased temperature, ocean acidity, hypoxia and hypercapnia killed many aquatic and terrestrial animals due to the abruptly reduced oxygen availability. A possibly related feature is the presence of the "fungal spike" in rocks near the Permian–Triassic boundary [33, 34].

It seems probable that on lowlands only small sized insects survived and the disappearance of giant insects made the spreading of fast-growing conifers feasible. New forests were soon repopulated by mammaliaformes that came down from several highland areas (*Lystrosaurs* might be an example [35]). Archosaurs were probably late in repopulation of the low lands from high mountain areas. Since they were adapted to very low pO_2_, archosaurs were not forced to descend to the sea level before the Triassic nadir value of 12% of relative oxygen content has been reached. During that devastating period, archosaurs prevailed in lowlands as only animals that could cope with profound hypoxia.

Some archosaurs, previously adapted to swimming in mountain rivers, probbaly reduced their oxygen demand by entering the low land rivers and became ancestors of crocodiles. Other archosaurs adapted to rapid climbing with improved cardiorespiratory functions and pneumatised bones. They became the ancestors of birds and pterosaurs. The remaining heavy built archosaurs also descended to the lowlands and became dinosaurs in Jurassic. It seems vary plausible that when the world has cooled and O_2_ level recovered, the highlands were also repopulated, this time by small archosaurs looking for places without large predators.

## Possible extrapolations

Additional file 2: Table S2 shows all mentioned points of animal evolution that were possibly related to the oxygen availability, suggesting that several great animal transitions and migrations might have been induced by reduced oxygen availability. After these disasters, the devastated biosphere was repeatedly repopulated from fringe habitats. On the other hand, periods of increased atmospheric oxygen often resulted in giant predators in lowland habitats, thus forcing smaller and weaker animals to live in relative safety of high mountains. All these topographic, altitude dependent biosphere fragmentations, defined by local oxygen and water availability, allowed separate evolution of small habitats for millions of years.

An obvious extrapolation of the presented interpretation regarding emergence of homeothermy as the prevalent metabolic mode of terrestrial and aerial animals is that the common ancestor of birds and non crocodilian archosaurs probably shared similar hearts, lungs and kidneys. If so, the full homeothermy is not expected to be present if these animals lived in warm habitats. Despite anatomic and physiological similarities of some dinosaurs with birds, it seems plausible that they did not need thermal insulation and stable endothermy. Instead of that, they required cooling mechanisms to dissipate heat after surges in energy expenditure.

Avian and mammalian sex chromosomes are important as determinations of inherited and thus temperature independent sex determination. After the K-Pg meteor impact, the birds and mammals with sex chromosomes reproduced well even in cold climates, while other animals with temperature dependent sex determination possibly failed, as has already been proposed [36, 37].

## Electronic supplementary material

Additional file 1: Table S1: Expected oxygen content in Devonian continental fresh water during daily heat and during the hypoxic Hangenberger event with an estimated drop in the atmospheric O_2_ content from 15 to 12% of O_2_. Values calculated from data available at: http://www.engineeringtoolbox.com/oxygen-solubility-water-d_841.html. (PDF 25 KB)

Additional file 2: Table S2: Schematic presentation of the proposed interpretation of pO_2_ driven animal evolution. Gray areas mark zones of different oxygen availability: zone A (dark) with pO_2_>220 mmHg supports giant insects & amphibians, zone B (medium) with 150<pO_2_<220 mmHg supports mammals, birds & small insects, zone C (light) with 90 < pO_2_ < 150 mmHg is mild hypoxia, and zone D (white) with pO_2_<90 mmHg is severe hypoxia. (PDF 23 KB)

Additional file 3: Table S3.: Schematic presentation of the proposed stratification of habitats according to the altitude. The values are calculated by the pO_2_ calculator, available at: http://www.altitude.org/air_pressure.php for different altitudes based on the average O2 content in that periods. Gray areas mark zones of different oxygen availability: zone A (dark) with pO_2_>220 mmHg supports giant insects & amphibians, zone B (medium) with 150<pO_2_<220 mmHg supports mammals, birds & small insects, zone C (light) with 90 < pO_2_ < 150 mmHg is mild hypoxia, and zone D (white) with pO_2_<90 mmHg is severe hypoxia. (PDF 17 KB)
