# Supplementary material for: Animal evolution and atmospheric pO2: is there a link between gradual animal adaptation to terrain elevation due to Ural orogeny and survival of subsequent hypoxic periods?
Source: Theor Biol Med Model. 2014 Oct 22;11:47. doi: 10.1186/1742-4682-11-47 (PMC4223737; doi:10.1186/1742-4682-11-47)
Supplement: Supplementary file 1 — Additional file 1: Table S1: Expected oxygen content in Devonian continental fresh water during daily heat and during the hypoxic Hangenberger event with an estimated drop in the atmospheric O2 content from 15 to 12% of O2. Values calculated from data available at: http://www.engineeringtoolbox.com/oxygen-solubility-water-d_841.html. (PDF 25 KB) [file 12976_2014_486_MOESM1_ESM.pdf]

Water temperature (℃)	Pure (100%) O_2 solubility in fresh water (air pressure 760 mmHg) (ml/L)	Devonian O_2 water content (air with 15% O_2)(ml/l)		Hangenberg event: O_2 content in water (est. 12% O_2 in air) (ml/l)	Δ of O_2 content during the Hangenberg event (air O_2 decrease from 15 to 12% O_2)(ml/l)
0	10.20	1.53	Δ of O_2 content for a 5℃ rise (ml/l)	1.22	-0.31
5	9.10	1.37	-0.17	1.09	-0.27
10	8.20	1.23	-0.14	0.98	-0.25
15	7.50	1.13	-0.11	0.90	-0.23
20	6.80	1.02	-0.11	0.82	-0.20
25	6.30	0.95	-0.08	0.76	-0.19
30	5.90	0.89	-0.06	0.71	-0.18
35	5.50	0.83	-0.06	0.66	-0.17
40	5.20	0.78	-0.05	0.62	-0.16
