# Supplementary material for: Animal evolution and atmospheric pO2: is there a link between gradual animal adaptation to terrain elevation due to Ural orogeny and survival of subsequent hypoxic periods?
Source: Theor Biol Med Model. 2014 Oct 22;11:47. doi: 10.1186/1742-4682-11-47 (PMC4223737; doi:10.1186/1742-4682-11-47)
Supplement: Supplementary file 2 — Additional file 2: Table S2: Schematic presentation of the proposed interpretation of pO2 driven animal evolution. Gray areas mark zones of different oxygen availability: zone A (dark) with pO2>220 mmHg supports giant insects & amphibians, zone B (medium) with 150<pO2<220 mmHg supports mammals, birds & small insects, zone C (light) with 90 < pO2 < 150 mmHg is mild hypoxia, and zone D (white) with pO2<90 mmHg is severe hypoxia. (PDF 23 KB) [file 12976_2014_486_MOESM2_ESM.pdf]

Age (Mya)	Geologic period/event	Oxygen content in the air	O_2 gradient and climate	Main land (mainly Pangea 300 to 200 Mya and later fragments)			
				Shallow waters	<500 m	500-1500 m above sea	>1500 m above sea
393.3-358.9	mid Devonian	15%	atmospheric pO2 rising above O_2 level in water	insect ancestors dwell beneath the water surface	high lignin plants produce large amount of O_2	hypoxia	
	late Devonian		high temperatures depleted O_2 from shallow waters	lobe-finned fish able to support themselves in water while breathing air	insects and high lignin plants		
358.9	Hangenberg hypoxic event	12%	drop in water oxygen forces amphibians out of water	extinction of many aquatic animals	early amphibians leave water	small amphibians & insects climb mountain slopes to escape predators	hypoxia
358.9-298.9	Carboniferous	32%	hyperoxia leads to giant animals in lowlands, small fringe habitats in highest mountains	various kinds of fish adapted to variable levels of water oxygenation	giant amphibians & insects, high lignin plants	**Ural orogeny (318-251 Mya)**	
	Late Carboniferous					first conifers small amphibians & insects, first reptiles mammaliaformes, ancestors of crocodiles,	diversification of archosaurs: ancestors of crocodiles, of dinosaurs archosaurs with unidirectional flow lungs developed to ancestors of birds & pterosaurs
298.9 to 252.2	Permian	mean at 23%	reduced oxygen level limits size of lowland insects		collapse of rainforests due to cooling and aridity		
252.28	P-Tr event	declining toward 16%	lignin decomposition by fungi increased CO2 and decreased O2	migration of crocodile ancestors to waters near the sea level	reduction in number of giant insects and amphibians	descent of all animals closer to the sea level due to hypoxia	hypoxia
252.2 to 201.3	Triassic	mean 16%, nadir at 12%	extinction of giant animals dependent on higher O2 content	spreading of crocodiles	extinction of giant insects, conifers prevail, reptiles survive, archosaurs repopulate land	dominance of conifers,	
