# Supplementary material for: Animal evolution and atmospheric pO2: is there a link between gradual animal adaptation to terrain elevation due to Ural orogeny and survival of subsequent hypoxic periods?
Source: Theor Biol Med Model. 2014 Oct 22;11:47. doi: 10.1186/1742-4682-11-47 (PMC4223737; doi:10.1186/1742-4682-11-47)
Supplement: Supplementary file 3 — Additional file 3: Table S3.: Schematic presentation of the proposed stratification of habitats according to the altitude. The values are calculated by the pO2 calculator, available at: http://www.altitude.org/air_pressure.php for different altitudes based on the average O2 content in that periods. Gray areas mark zones of different oxygen availability: zone A (dark) with pO2>220 mmHg supports giant insects & amphibians, zone B (medium) with 150<pO2<220 mmHg supports mammals, birds & small insects, zone C (light) with 90 < pO2 < 150 mmHg is mild hypoxia, and zone D (white) with pO2<90 mmHg is severe hypoxia. (PDF 17 KB) [file 12976_2014_486_MOESM3_ESM.pdf]

**Altitude above the sea (m)**	**Atmospheric pressure (mmHg)**	**O_2 availability (%) & terrestrial life**	**pO_2 (mmHg)**							
			Devonian (15% O_2)	Carboniferous (32.5% O_2)	Permian (23% O_2)	P-Tr extinction (16% O_2)	Triassic nadir (12% O_2)	Jurassic (26% O_2)	Modern (21% O_2)	Topography
0	760	100	114.0	247.0	174.8	121.6	91.2	198.0	159.6	Lowland forests
500	714.4	94	107.2	232.2	164.1	114.3	85.7	186.0	150.0	Hills
1000	676.4	89	101.5	219.8	155.6	108.2	81.2	176.0	142.0	Low mountains
1500	638.4	84	95.8	207.5	146.8	102.1	76.6	166.0	134.1	High mountains (mainly Ural orogeny)
2000	615.6	81	92.3	200.1	141.6	98.5	73.9	160.0	129.3	
2500	570	75	85.5	185.3	131.1	91.2	68.4	148.0	119.7	
Possibly related events				Ural orogeny slowly raised few mountain habitats to >2000 m above sea,		South China Craton (SCC) moved north from equator		SCC collided with Siberia		
